# Quantitative Measurement of the Affinity of Toxic and Nontoxic Misfolded Protein Oligomers for Lipid Bilayers and of its Modulation by Lipid Composition and Trodusquemine

**DOI:** 10.1021/acschemneuro.1c00327

**Published:** 2021-08-12

**Authors:** Silvia Errico, Hassan Ramshini, Claudia Capitini, Claudio Canale, Martina Spaziano, Denise Barbut, Martino Calamai, Michael Zasloff, Reinier Oropesa-Nuñez, Michele Vendruscolo, Fabrizio Chiti

**Affiliations:** † Department of Experimental and Clinical Biomedical Sciences, Section of Biochemistry, University of Florence, Florence 50134, Italy; ‡ Centre for Misfolding Diseases, Yusuf Hamied Department of Chemistry, University of Cambridge, Cambridge CB2 1EW, United Kingdom; § Department of Biology, Payame Noor University, Tehran 19395-4697, Islamic Republic of Iran; ∥ European Laboratory for Non-linear Spectroscopy (LENS), Sesto Fiorentino 50019, Italy; ⊥ Department of Physics and Astronomy, University of Florence, Sesto Fiorentino 50019, Italy; # Department of Physics, University of Genoa, Genoa 16146, Italy; ∇ Enterin Inc., 2005 Market Street, Philadelphia, Pennsylvania 19103, United States; ○ National Institute of Optics, National Research Council of Italy (CNR), Florence 50125, Italy; ◆ MedStar-Georgetown Transplant Institute, Georgetown University School of Medicine, Washington D.C. 20007, United States; + Department of Materials Science and Engineering, Uppsala University, Uppsala SE-751 03, Sweden

**Keywords:** Alzheimer’s disease, Parkinson’s disease, protein misfolding, neurodegeneration, aminosterols, squalamine

## Abstract

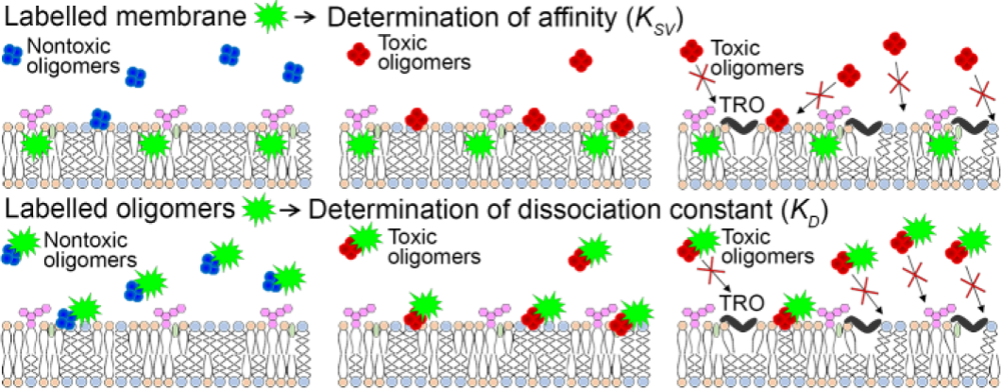

Many neurodegenerative diseases are associated with the self-assembly of peptides and proteins into fibrillar aggregates. Soluble misfolded oligomers formed during the aggregation process, or released by mature fibrils, play a relevant role in neurodegenerative processes through their interactions with neuronal membranes. However, the determinants of the cytotoxicity of these oligomers are still unclear. Here we used liposomes and toxic and nontoxic oligomers formed by the same protein to measure quantitatively the affinity of the two oligomeric species for lipid membranes. To this aim, we quantified the perturbation to the lipid membranes caused by the two oligomers by using the fluorescence quenching of two probes embedded in the polar and apolar regions of the lipid membranes and a well-defined protein–oligomer binding assay using fluorescently labeled oligomers to determine the Stern–Volmer and dissociation constants, respectively. With both approaches, we found that the toxic oligomers have a membrane affinity 20–25 times higher than that of nontoxic oligomers. Circular dichroism, intrinsic fluorescence, and FRET indicated that neither oligomer type changes its structure upon membrane interaction. Using liposomes enriched with trodusquemine, a potential small molecule drug known to penetrate lipid membranes and make them refractory to toxic oligomers, we found that the membrane affinity of the oligomers was remarkably lower. At protective concentrations of the small molecule, the binding of the oligomers to the lipid membranes was fully prevented. Furthermore, the affinity of the toxic oligomers for the lipid membranes was found to increase and slightly decrease with GM1 ganglioside and cholesterol content, respectively, indicating that physicochemical properties of lipid membranes modulate their affinity for misfolded oligomeric species.

## Introduction

Many neurodegenerative diseases, including Alzheimer’s disease (AD), Parkinson’s disease (PD), Creutzfeld-Jacob disease (CJD), Huntington disease (HD), frontotemporal dementia (FTD), familial amyloid polyneuropathy (FAP), and many others are associated with the self-assembly of specific peptides or proteins into misfolded fibrillar aggregates known as amyloid fibrils.^1,2^ The formation of amyloid fibrils is a generic characteristic of proteins, and it is also found for a large number of systems that are not associated with disease.^3−5^ Multiple lines of evidence suggest that small soluble oligomers formed in the process of amyloid fibril formation or released by mature fibrils are important players of the neurotoxicity associated with protein aggregation.^1,6−10^ These species have an ability to bind to and destabilize biological membranes, inducing an entry of Ca^2+^ from the extracellular space into the cytosol. This phenomenon seems to be generic, as it has been found for oligomers of the amyloid β peptide,^11−13^ α-synuclein,^9,14^ islet-amyloid polypeptide,^15^ calcitonin,^16,17^ as well as model oligomers.^11,18,19^

The study of the structural elements of misfolded protein oligomers responsible for neuronal dysfunction and of the mechanism through which they cause neurotoxicity has benefited from the isolation of pairs of oligomers of the same protein having a toxic and benign effect, respectively.^18−23^ One of such pairs is that obtained from the 91-residue N-terminal domain of [NiFe]-hydrogenase maturation factor HypF (HypF-N) from *E. coli*.^18,19^ Oligomer formation of HypF-N can be readily and reproducibly directed into two morphologically similar forms, previously named type A (toxic) and type B (nontoxic) oligomers (OAs and OBs, respectively), by altering the solution conditions. Although this protein domain has no known link to human disease, it has proved to be a valuable model system as its toxic OAs have been shown to have effects indistinguishable from those of Aβ_42_ oligomers associated with AD at the biochemical, electrophysiological and animal model levels.^11,18,19,24−29^

OAs manifest a strong ability to bind and penetrate the lipid bilayer of cell membranes.^18,19,30^ This interaction induces a series of downstream events associated with cytotoxicity, including an influx of calcium ions (Ca^2+^) from the cell medium to the cytoplasm, generation of intracellular reactive oxygen species (ROS), lipid peroxidation, perforation of cell membranes with exit of intracellularly trapped large molecules, caspase-3 activation, and mitochondrial damage.^18,19^ OAs also induce loss of cholinergic neurons when microinjected into rat brains with an associated impairment of spatial memory in rats, colocalization with synapses in primary neurons, and inhibition of long-term potentiation (LTP) in rat hippocampal slices.^19,26^ By contrast, OBs were found to bind to lipid membranes on the surface, without infiltrating their lipid bilayer, and were not found to have any of the deleterious biological effects observed for OAs.^18,19,26^

A recent interactome approach to detect all the membrane proteins of microglia N13 cells interacting with OAs and OBs showed that OBs had a higher affinity for membrane proteins relative to OAs.^31^ By contrast, other experiments with supported lipid bilayers (SLBs) devoid of proteins treated with OAs and OBs and monitored with atomic force microscopy (AFM) revealed that numerous OAs were bound strongly with both the gel and liquid-disordered phases of SLBs, whereas few OBs were found to have this ability.^30^ It was therefore concluded that although both species bind to cell membranes, only OAs bind to, penetrate, and cross the lipid bilayer of the membranes, thus manifesting their toxic behavior.^30,31^ In particular, the GM1 concentration dependence of the OA toxicity in cell cultures and of OA binding to SLBs showed that the oligomers binding to the gel-phase (L_β_ or S_o_) of the lipid bilayer are responsible for the OA toxicity.^30^

In terms of their morphology and structure, both oligomer types were found to be highly stable, spheroidal, or discoidal under atomic force microscopy (AFM) with a diameter of 2–6 nm, to possess a similar β-sheet core structure, and to display weak thioflavin T (Th-T) binding.^18,32,33^ In spite of these structural similarities, characterization of these species at a molecular level revealed important differences.^18,32^ By labeling the oligomers with pyrene at various sequence positions, it was shown that nontoxic OBs are stabilized by intermolecular interactions between the three major hydrophobic regions of the sequence, such that a lower fraction of the hydrophobic residues are solvent-exposed on the oligomer surface relative to the toxic OA species.^18^ Such interactions are weaker in the toxic oligomers so that a larger fraction of hydrophobic residues are solvent exposed.^18^ The binding of the fluorescent reporter 8-anilinonaphthalene-1-sulfonic acid (ANS) was also found to be higher in OAs, confirming a higher exposure of hydrophobic clusters.^18^ Solution-state and solid-state nuclear magnetic resonance (NMR) spectroscopy and site-directed fluorescence resonance energy transfer (FRET) experiments, involving residues labeled with a donor and acceptor at various positions, showed that toxic OAs have a greater compactness and structural rigidity, so that structural constraints are generated that cause a number of the hydrophobic residues to interact less strongly with each other, with a fraction of them becoming exposed to the solvent.^32^ Accordingly, FRET efficiency values were, on average, higher in toxic OAs than nontoxic OB species, except when donor and acceptor labeling involved hydrophobic residues, when the opposite situation was observed.^32^

The binding of OAs to the bilayer of cell membranes represents an important event in the mechanism through which these oligomeric species manifest their toxicity to neuroblastoma cells and primary neurons.^11,18,19^ However, given the complexity of cellular systems, methods for quantitative measurements of oligomer-membrane binding, for example, in terms of association or dissociation constants, are not well established. In addition, it is not clear whether the oligomers change their structures upon interacting with cell membranes. To address these issues, we have used liposomes in the form of large unilamellar vesicles (LUVs) with a variable and biologically compatible lipid composition and OAs/OBs of HypF-N. Our results indicate that OAs are characterized by a higher and measurable affinity than OBs for the LUV bilayer and that the native protein does not bind to the lipid membranes. We also found that neither oligomer type changes its structure upon interaction with the LUVs and that OAs do not feature a preferential binding to any of the lipids contained in LUVs. We then reveal quantitatively the effects of trodusquemine, a promising and previously studied small molecule that binds to the membrane, on the OA–membrane affinity and on the mechanism of displacement of these toxic oligomers from the bilayer and, again in a quantitative manner, how the lipid composition of LUVs can influence the affinity of toxic oligomers for the membranes.

## Results and Discussion

### The Binding Affinity of OAs to LUVs is 20–25 Times Higher than That of OBs

Toxic OAs and nontoxic OBs were preformed from purified HypF-N at a total concentration of 0.5 mg/mL, corresponding to 48 μM (monomer equivalents), as previously reported.^18,33^ LUVs were prepared using DOPC and SM in a molar ratio of 2:1 (mol/mol), 1% (mol) CHOL and 1% (mol) GM1, as previously reported.^34^ LUVs were prepared at various mass concentrations (mg/mL); at a total lipid concentration of 1 mg/mL, for example, molar concentrations were 836 μM DOPC, 418 μM SM, 13 μM CHOL and 13 μM GM1.

We first checked whether OAs and OBs bound to LUVs. To this purpose, we formed separately supported lipid bilayers (SLBs) with the same lipid composition as LUVs, treated them with 12 μM OAs or 12 μM OBs (monomer equivalents), and then imaged them with AFM. The images show that OAs bind to the gel-phase domains (L_β_ or S_o_) and to the liquid-disordered phase (L_α_ or L_d_) of the SLBs with 1% GM1, whereas only few OBs were found to be bound to them (Figure S1), in agreement with previous results obtained with 5% GM1 as the only difference in LUV composition relative to our LUV preparations.^30^ Furthermore, the difference in the thickness between the L_β_ and L_α_ domains (Δ*Z*) is altered by the presence of OAs, but not OBs, clearly indicating the presence of structural changes of the overall bilayer (Table S1), again in agreement with the result obtained with 5% GM1.^30^

**Figure 1:**
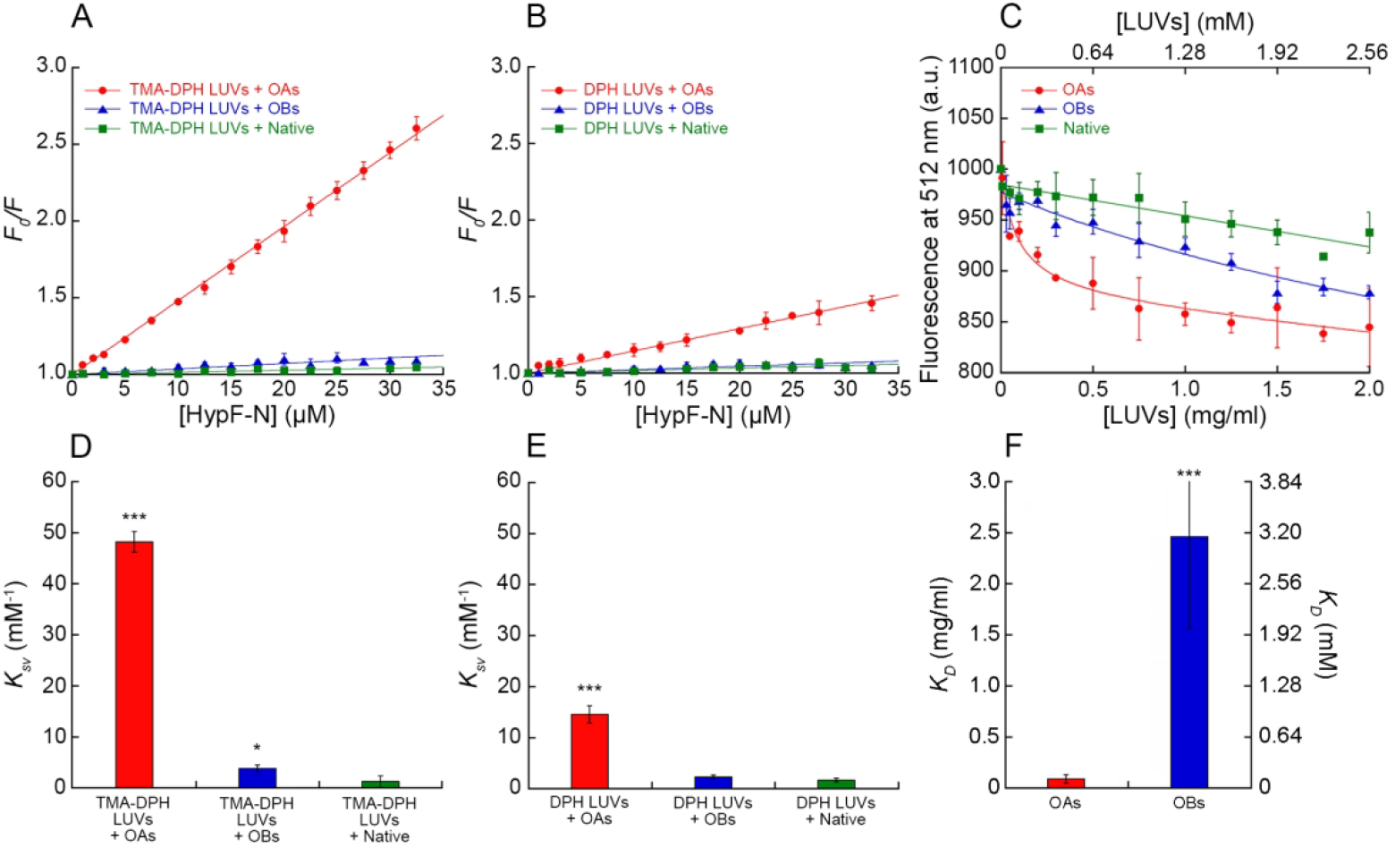
Binding of OAs/OBs/native HypF-N to LUVs. (A,B) Stern–Volmer plots reporting the ratio of fluorescence of TMA-DPH (A) and DPH (B) in 0.3 mg/mL LUVs in the absence (*F*_0_) or presence (*F*) of various concentrations (monomer equivalents) of OAs (red circles), OBs (blue triangles), and native HypF-N (green squares). The straight lines through the data points represent the best fits to eq 4. (C) Binding plots reporting the fluorescence at 512 nm of 20 μM BODIPY-FL-labeled OAs (red circles), OBs (blue triangles), and native HypF-N (green squares) versus LUV concentration reported in mg/mL units (bottom *x* axis) or mM units (top *x* axis). The lines through the data points represent the best fits to eq 6. (D,E) Bar plots reporting the *K*_SV_ values obtained from TMA-DPH (D) and DPH (E) fluorescence quenching using eq 4. (F) Bar plots reporting the *K*_D_ values from binding using eq 6. Experimental errors represent SEM of 2–5 experiments. The symbols * and *** refer to *p* values of <0.1 and <0.001, respectively, relative to *K*_SV_ values of the native protein (D,E) and relative to the *K*_D_ value of OAs (F).

In order to obtain a more quantitative measure of the binding affinity of the OAs and OBs for lipid membranes, we evaluated the ability of these oligomers to quench DPH and its derivative TMA-DPH, two fluorescent probes that incorporate within the hydrophobic region^35^ and polar head region^36^ of the lipid bilayer, respectively. The 15 min incubation of increasing concentrations of OAs (from 0 to 32.5 μM monomer equivalents) with TMA-DPH- and DPH-labeled LUVs (0.3 mg/mL, 384 μM total lipids) caused a marked and concentration-dependent reduction of the fluorescence emission of both fluorescent probes, with a more consistent quenching of TMA-DPH (Figure 1A,B). The *K*_SV_ constant is a measure of the quenching of the dye fluorescence operated by the oligomers and obtained by fitting the data to eq 4 (see Materials and Methods) and is also a measure of the affinity of the oligomers for the membrane-embedded probe, as it reports on the collisions between OAs and the probe.^37^ The *K*_SV_ value was found to be 48.2 ± 2.0 mM^–1^ for TMA-DPH and 14.5 ± 1.7 mM^–1^ for DPH (Figure 1). The incubation of OBs with TMA-DPH- and DPH-labeled LUVs under identical conditions caused a significantly weaker fluorescence quenching (Figures 1A,B and S2A,B), with *K*_SV_ values of 3.7 ± 0.7 and 2.2 ± 0.4 mM^–1^, respectively, which reflected a lower binding to the membrane and the absence of lipid membrane alteration (Figure 1). Native HypF-N showed a substantially absent ability to quench both TMA-DPH and DPH (Figures 1A,B and S2A,B), with *K*_SV_ values of 1.2 ± 1.1 mM^–1^ and 1.6 ± 0.4 mM^–1^, respectively, reflecting the absence of binding to the membrane (Figure 1). By subtracting these two background values from the corresponding ones obtained for OAs and OBs, one can determine that OAs have *K*_SV_ values ca. 20-fold higher than OBs with both probes.

To obtain an independent measure of the binding affinity of the three HypF-N species for the LUV membrane, we labeled HypF-N with BODIPY FL and then prepared samples of OAs, OBs and native proteins using the labeled and unlabeled protein at a molar ratio of 1:10. The 15 min incubation of native HypF-N (20 μM) with increasing concentrations of unlabeled LUVs (0–2.0 mg/mL, 0–2.6 mM total lipids) caused a weak decrease of protein fluorescence that was found to correlate linearly with LUV concentration (Figure 1C). A similar decrease, even with the same slope, was observed for the highly soluble reduced glutathione (GSH) labeled with BODIPY FL (Figure S3), indicating that it consists of a LUV-induced fluorescence drift, most probably arising from light scattering as the LUV concentration increases. The 15 min incubation of OAs (20 μM monomer equivalents) with increasing concentrations of unlabeled LUVs (0–2.0 mg/mL, 0–2.6 mM) caused a marked decrease of OA fluorescence from 0 to *ca*. 0.4 mg/mL LUVs (corresponding to 0.5 mM total lipids), followed by the same drift at higher LUV concentrations (Figure 1C). By fitting the data points to a binding function (eq 6), we obtained a dissociation constant (*K*_D_) value of 0.09 ± 0.04 mg/mL, corresponding to 0.12 ± 0.05 mM of total lipids, indicating binding of OAs to the LUV bilayer. The fluorescence of OBs also decreased significantly with LUV concentration, to an extent lower relative to that of OAs, but larger relative to native HypF-N or GSH (Figure 1C), indicating real binding. The fitting of the data points to eq 6 led to a *K*_D_ value of *ca*. 2.5 mg/mL, corresponding to ca. 3.2 mM of total lipids, indicating an affinity for LUVs lower, by ca. 25-fold, relative to OAs.

Hence, under the conditions used here, we have quantified the binding affinity of toxic OAs and nontoxic OBs of a sample protein for the bilayer of lipid vesicles (LUVs) by measuring the *K*_SV_ values of fluorescence quenching of membrane-embedded TMA-DPH and DPH caused the oligomers (0.3 mg/mL LUVs or 384 μM total lipids) and the oligomer-membrane *K*_D_ values (20 μM protein in monomer equivalents). Although *K*_SV_^–1^ has been shown to correspond to *K*_D_,^37^ these values are not immediately comparable in our system because they were measured in different conditions, that is at constant LUV concentration (0.3 mg/mL) upon varying OA concentration and at constant OA concentration (20 μM), upon varying that of LUVs, respectively. The molarities of *K*_SV_^–1^ and *K*_D_ also refer to protein and total lipids, respectively, and are not, therefore, comparable. In both cases, however, the binding affinity of the toxic OAs for the membrane appears to be ca. 20–25 times higher than nontoxic OBs. Albeit with much lower affinity, nontoxic OBs also bind to the LUV membrane, unlike the native protein.

Can we relate the data obtained here with LUVs to cell cultures and brain tissues? By using a mean diameter of 7.5 ± 0.5 μm known for human neuroblastoma SH-SY5Y cells,^38^ a lipid density of a membrane bilayer estimated from LUVs of 425 ± 3 ng/cm^2^,^34^ and a cell density value of neuroblastoma SH-SY5Y cells commonly used to test the toxicity of OA/OB species of *ca*. 7.5(±0.7) 10^4^ cells/cm^2^,^29^ and then extrapolating this value to the three-dimensional space, one can determine a lipid concentration of 0.06 ± 0.01 mg/mL in SH-SY5Y cell cultures where OA/OB species are tested. The *K*_D_ value of 0.09 ± 0.04 mg/mL lipids measured here for OAs, and referred to total lipid concentration, implies that a significant fraction of OAs (40 ± 15%) are bound to the lipid membranes of cells, as soon as the equilibrium between membrane-bound and membrane-unbound OAs is established and before they enter into the cells. By contrast, the *K*_D_ value of ∼2.5 mg/mL lipids measured for OBs, implies that a very minor fraction of OB species interact with the cell membrane (∼2%).

### The Binding to LUVs Does Not Detectably Affect the Structures of OAs and OBs

One of the questions that is often raised when studying the structure–toxicity relationship of misfolded protein oligomers is whether the structural characteristics determined for the oligomers in aqueous suspension are maintained or changed upon the interaction with biological membranes. Difficulties to address this issue arise from interferences by cellular or membrane proteins that make it very difficult to monitor the structural characteristics of the oligomers with conventional spectroscopic probes. Here we circumvented this problem using protein-free LUVs and three optical probes to which LUVs are spectroscopically silent, making it possible to monitor the secondary and tertiary structure of the oligomers before and after their binding to the membrane.

**Figure 2:**
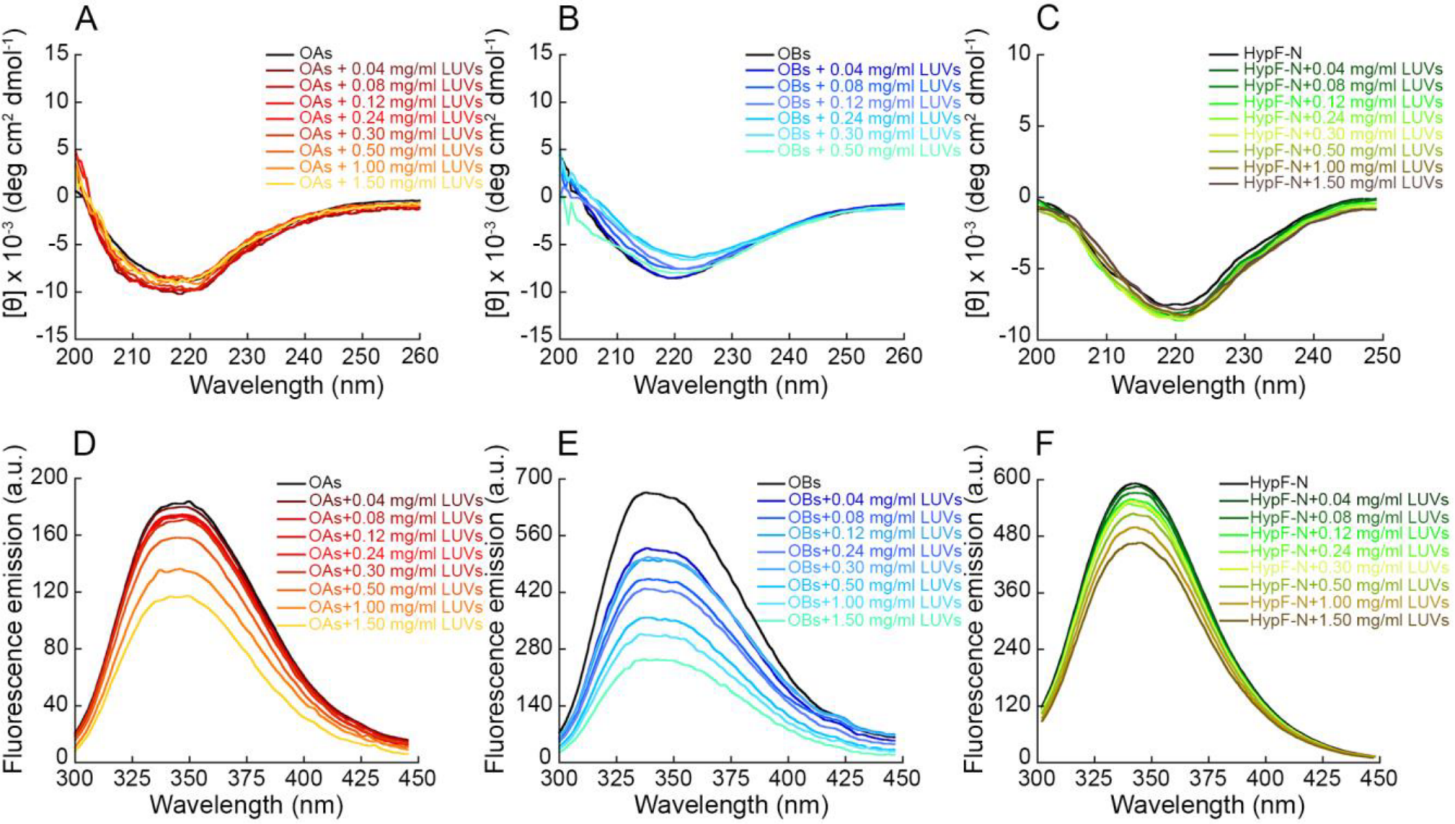
Far-UV CD and intrinsic fluorescence spectra of OAs, OBs, and native HypF-N. (A–C) Far UV CD spectra of OAs (A), OBs (B), and native HypF-N (C) in the presence of increasing concentrations of LUVs. Spectra were blank-subtracted and normalized using eq 1. (D–F) Intrinsic tryptophan fluorescence emission spectra of OAs (D), OBs (E), and native HypF-N (F) in the presence of increasing concentrations of LUVs.

We first acquired far-UV CD and intrinsic fluorescence spectra of OAs, OBs and native HypF-N incubated with increasing concentrations of LUVs. The far-UV CD spectra of OAs, OBs, and native HypF-N (20 μM monomer equivalents) were not found to be significantly different in the absence or presence of the various LUV concentrations (0–1.5 mg/mL), indicating that their secondary structure was maintained upon interaction with LUVs (Figure 2A–C). The intrinsic tryptophan fluorescence spectra of the three species (1.9 μM monomer equivalents) were also similar in the absence or presence of the various LUV concentrations (0–1.5 mg/mL) in terms of wavelength of maximum fluorescence and overall shape, featuring only a linear intensity decrease as the LUV concentration increases, again attributable to light scattering caused by LUVs, as explained above. This indicates that the presence of LUVs did not influence the chemical environment around the tryptophan residues of the protein (Figure 2D–F).

**Figure 3:**
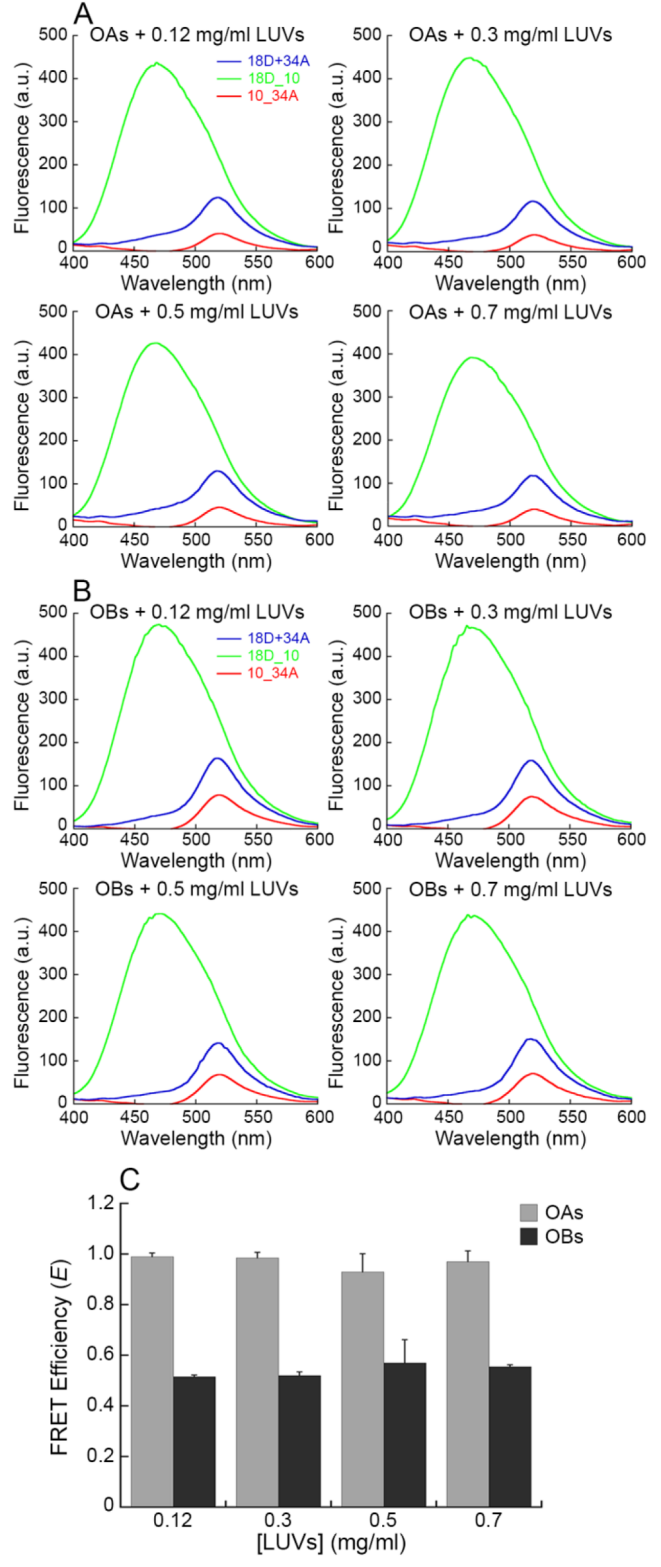
Intraoligomer FRET between OAs and OBs LUVs. (A,B) Fluorescence emission spectra of OAs (A) and OBs (B) formed by 18D_10 (green), 10_34A (red), and 18D+34A (blue), obtained in the presence of increasing concentrations of LUVs (0.12, 0.3, 0.5, and 0.7 mg/mL). (C) FRET *E* values of OAs (gray) and OBs (black) in the presence of increasing concentrations of LUVs, determined using eq 2. Experimental errors are SD.

In addition, we performed experiments of intraoligomer FRET in the presence of increasing concentrations of LUVs. Two HypF-N mutants having only one cysteine residue at positions 18 and 34 were labeled with the donor dye 1,5-IAEDANS and the acceptor dye 6-IAF, respectively, and then mixed in a 1:1 molar ratio to form OAs and OBs to a final protein concentration of 20 μM monomer equivalents. The FRET *E* values determined by analyzing the resulting fluorescence spectra acquired following 10 min-incubation with unlabeled LUVs (0–0.7 mg/mL) were not found to significantly change when varying LUV concentration, either for OAs or for OBs (Figure 3). This result indicates that the mean shortest donor–acceptor distance in the oligomers does not change upon LUV addition and suggests that the intermolecular structure of the oligomers was not significantly altered by the presence of LUVs.

### The OA-LUV Binding Does Not Involve Specific Lipid Species

**Figure 4:**
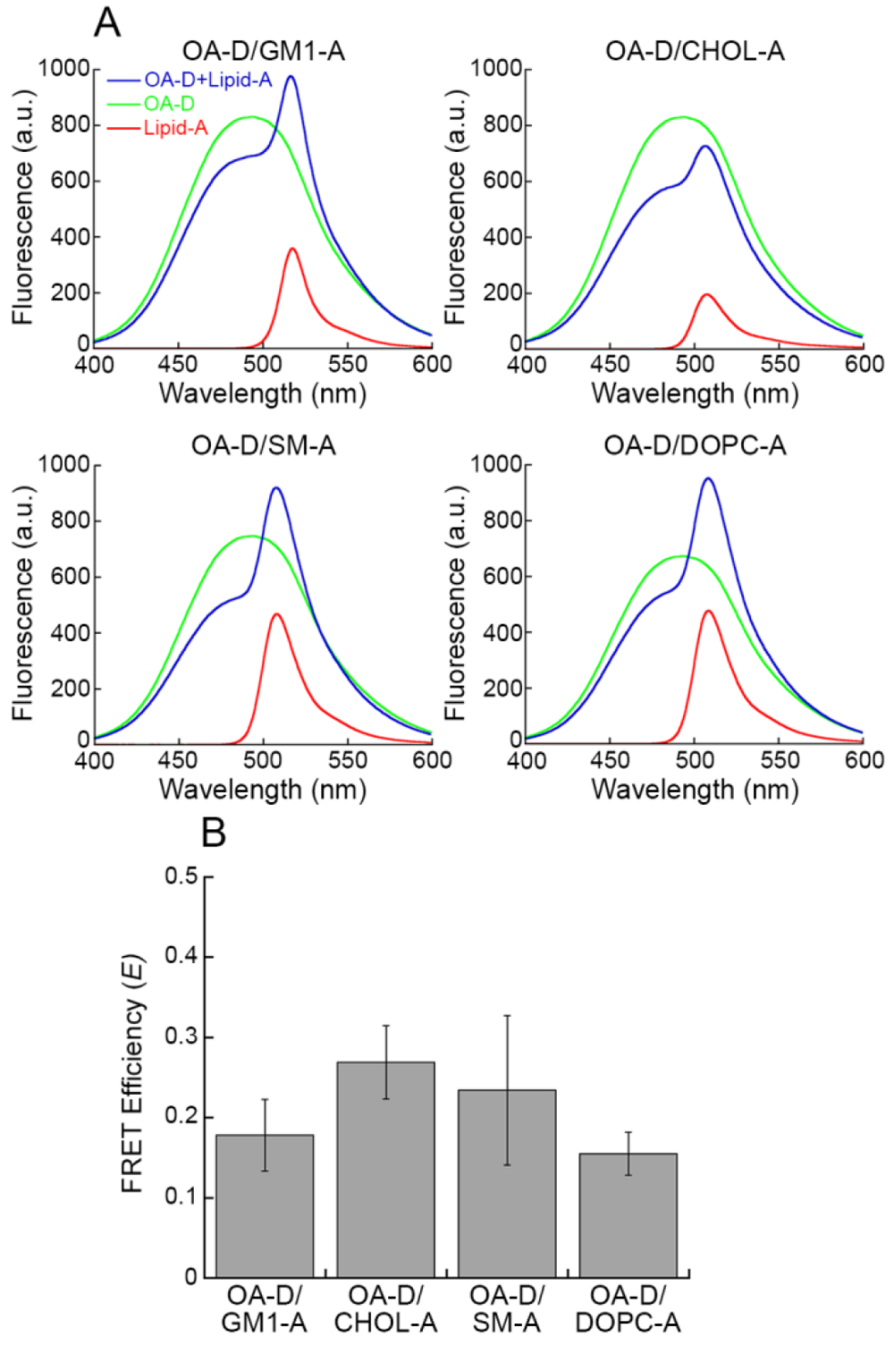
FRET between OAs labeleld with donor (D) and the various lipids labeled with acceptor (A) contained in LUVs. (A) Fluorescence emission spectra of OA-D+Lipid-A (blue), OA-D (green), and Lipid-A (red). (B) FRET *E* values of the indicated FRET pairs examined, obtained using eq 3. Experimental errors represent SEM of 5 experiments.

Since only OAs were found to have a high affinity for LUVs, we continued our study with this species. In order to investigate whether the binding between LUVs and OAs could depend on a specific interaction with one of the lipids contained in LUVs, we performed FRET experiments using 20 μM (monomer equivalents) OAs labeled with 1,5-IAEDANS as a donor probe (OA-D) and 0.3 mg/mL LUVs containing one of the four lipids labeled with BODIPY-FL as an acceptor probe (Lipid-A). These experiments were carried out separately by using each of four lipids contained in the LUVs labeled with A. The FRET *E* values were obtained by the analysis of the fluorescence spectra acquired after 15 min (Figure 4A) and were found to be 0.18 ± 0.04 for OA-D/GM1-A, 0.27 ± 0.05 for OA-D/CHOL-A, 0.23 ± 0.09 for OA-D/SM-A and 0.15 ± 0.03 for OA-D/DOPC-A, without significant differences between the various FRET pairs examined (Figure 4B).

This analysis indicates that OAs bind to LUVs but do not have a preferential interaction with any of the four lipids. Therefore, the role played by GM1 in the oligomer–membrane interaction, observed here and previously,^11,30,39^ involves a change of the bilayer physical properties, without a direct preferential interaction of the lipid with the oligomers. Indeed, it is clear that GM1 increases the overall negative net charge of the membrane,^34^ increases the thickness of the membrane, particularly of the L_β_ phase,^30,40^ and decreases lateral diffusion,^41^ all known to contribute to a facilitated oligomer insertion.

### Trodusquemine Reduces the Binding Affinity of OAs for LUVs

**Figure 5:**
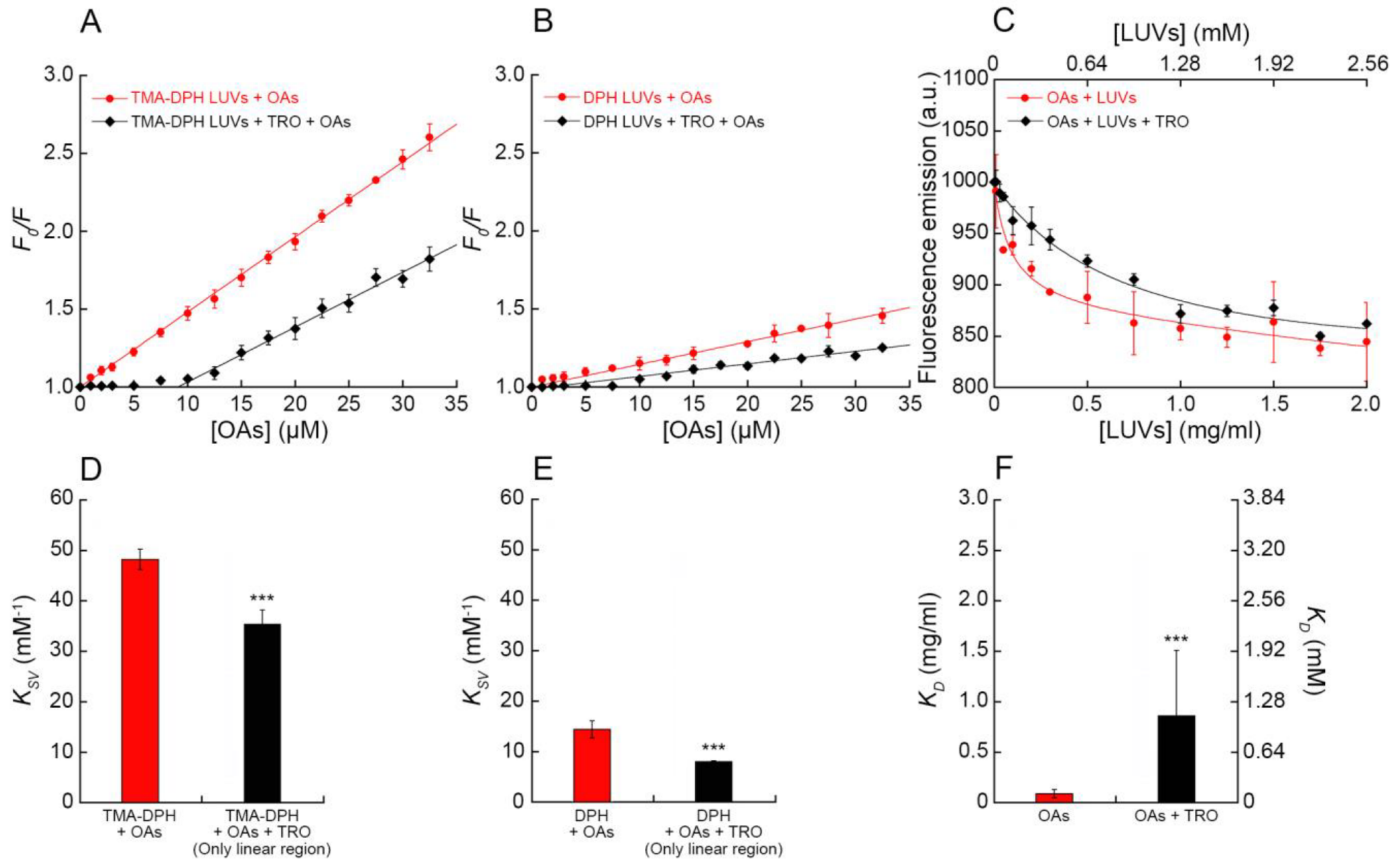
Interaction of OAs with LUVs with and without trodusquemine. (A,B) Stern–Volmer plots reporting the ratio of fluorescence of TMA-DPH (A) and DPH (B) in the absence (*F*_0_) or presence (*F*) of various concentrations (monomer equivalents) of OAs, in the absence (red circles) and presence (black diamonds) of 5 μM trodusquemine (TRO) in 0.3 mg/mL LUVs. The straight lines through the data points represent the best fits to eq 4 (red line) and eq 5 (black line). Experimental errors represent SEM of 2–5 experiments. (C) Binding plots reporting the fluorescence at 512 nm of OAs in the absence (red circles) and presence (black diamonds) of TRO in LUVs, versus LUV concentration. The lines through the data points represent the best fits to eq 6. (D,E) Bar plots reporting the *K*_SV_ values obtained from TMA-DPH (D) and DPH (E) fluorescence quenching in the absence (red) and presence (black) of 5 μM trodusquemine. (F) Bar plots reporting the *K*_D_ values obtained from the binding experiments of OAs in the absence (red) and presence (black) of 5 μM trodusquemine. Experimental errors represent SEM of 2–5 experiments. The symbols *** refer to *p* values of <0.001 relative to *K*_SV_ (D,E) and *K*_D_ (F) values of OAs in the absence of trodusquemine.

We then investigated whether trodusquemine (Figure S4), which has been reported to displace toxic oligomers from lipid membranes,^27,42,43^ could induce a variation of the affinity of OAs for the membrane, as measured with TMA-DPH and DPH fluorescence quenching and OA fluorescence change upon LUV binding. To this aim, we prepared TMA-DPH and DPH-labeled LUVs (0.3 mg/mL, 384 μM total lipids) containing 5 μM trodusquemine, and we incubated them with increasing concentrations of unlabeled OAs (0–32.5 μM monomer equivalents). Previous experiments have shown that trodusquemine has a high affinity for LUVs of this type and partitions completely in the bilayer at this concentration.^34^ The presence of trodusquemine in the bilayer caused a significant reduction of the TMA-DPH fluorescence quenching, with an almost complete absence of quenching at low concentrations of OAs up to ca. 10 μM (Figure 5A). At higher concentrations of OAs, the TMA-DPH quenching was evident, but remained lower than that observed in the absence of trodusquemine at corresponding OA concentrations, showing a reduction of the affinity of OAs for LUVs (Figure 5A). A similar profile was observed by repeating the experiment with DPH-labeled LUVs (Figures 5B and S2C).

Interestingly, the absence of TMA-DPH and DPH fluorescence quenching at the concentrations of OAs that normally cause dysfunction and toxicity to cell cultures (<10 μM monomer equivalents) and at the concentration of trodusquemine that causes protection (5 μM) indicates that this small molecule provides protection by preventing OA-LUV binding. By contrast, at higher OA concentrations, the oligomer-displacing effect of the molecule is overcome, most probably because in the excess of OAs trodusquemine partitions to OAs more markedly^27^ and populates the membrane to a lower extent. However, under these excess OA concentrations and in the presence of 5 μM trodusquemine, the *K*_SV_ constant remains lower than that observed in the absence of the small molecule. This phenomenon indicates that at the toxic OA concentrations and protective trodusquemine concentrations the molecule is largely effective as a protective factor but partly loses its protective action in the presence of excess oligomers.

We then incubated BODIPY FL-OAs (20 μM) with increasing concentrations of unlabeled LUVs (0–2.0 mg/mL, 0–2.6 mM total lipids) containing the same molar fraction of trodusquemine, and we repeated the analysis described above in the absence of the small molecule, but this time with the molecule (Figure 5C). Trodusquemine was found to significantly increase the *K*_D_, from a value of 0.09 ± 0.04 mg/mL in its absence (corresponding to 0.12 ± 0.05 mM lipids) to a value of 0.86 ± 0.65 mg/mL in its presence (corresponding to 1.10 ± 0.83 mM lipids), therefore reducing the binding affinity of OAs to LUVs by 1 order of magnitude (Figure 5C). Using the same arguments described above to translate these data into a cell culture context, under these conditions of analysis trodusquemine induces a decrease of membrane-bound OAs from ∼40% in the absence of the molecule to ∼6% in its presence. Numerical values of *K*_SV_ and *K*_D_ with and without trodusquemine are reported in Figure 5D–F.

### Change of OA-LUV Binding Affinity with LUV Composition

**Figure 6:**
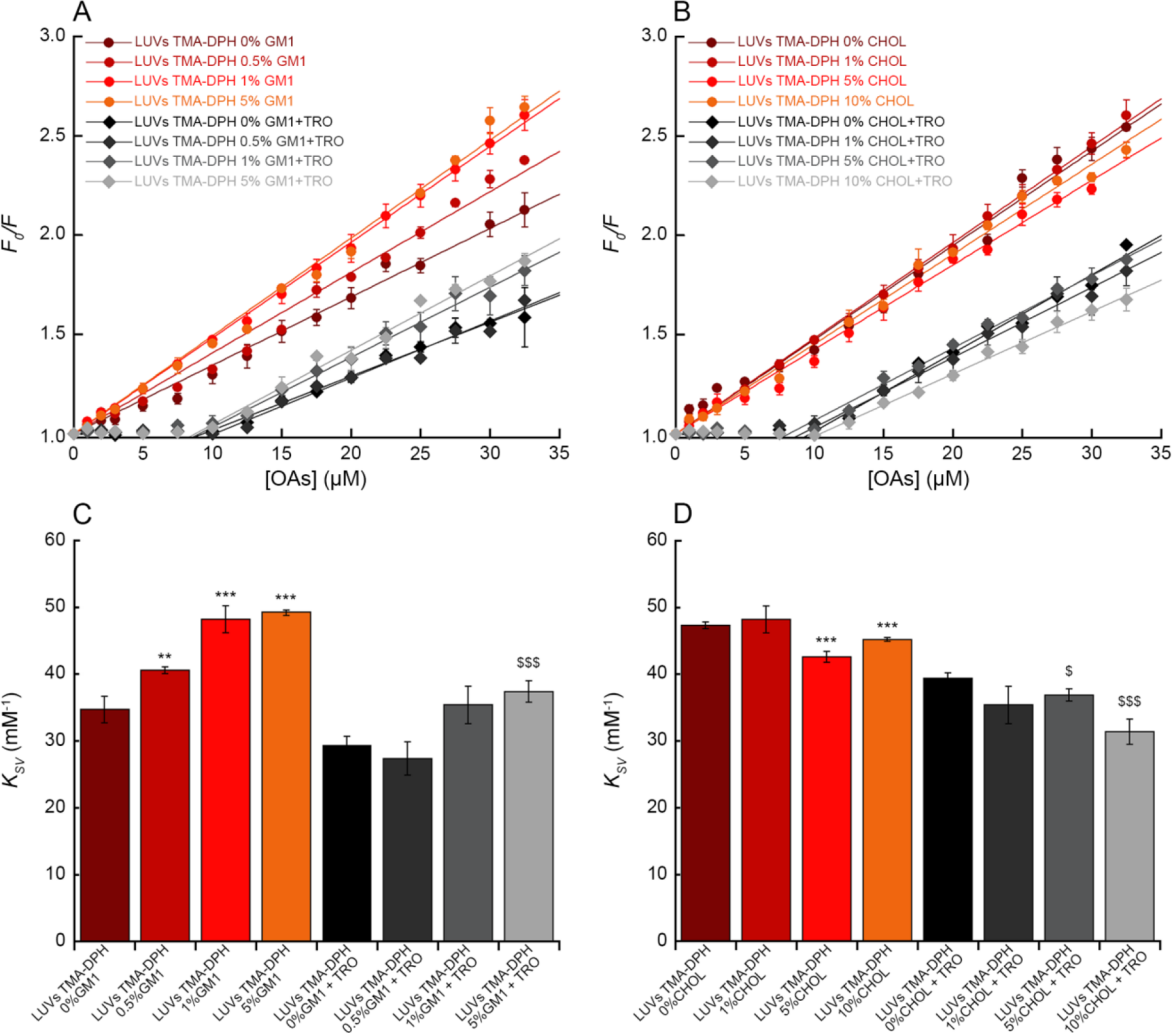
Binding of OAs to LUVs with various lipid compositions. (A,B) Stern–Volmer plots reporting the ratio of fluorescence of TMA-DPH in the absence (*F*_0_) or presence (*F*) of various concentrations (monomer equivalents) of OAs, in the absence (various shades of red circles), and in the presence (various shades of gray diamonds) of 5 μM trodusquemine (TRO) in 0.3 mg/mL LUVs containing different percentage of GM1 (A) and CHOL (B). The straight lines through the data points represent the best fits to eq 4 (various shades of red lines) and eq 5 (various shades of gray lines). (C,D) Bar plots reporting the *K*_SV_ values obtained from TMA-DPH fluorescence quenching in LUVs containing different percentages of GM1 (C) and CHOL (D) in the absence (various shades of red) and in the presence (various shades of gray) of 5 μM trodusquemine. Experimental errors represent SEM of 2–5 experiments. The symbols ** and *** refer to *p* values of <0.01 and <0.001, respectively, relative to *K*_SV_ values of OAs without GM1 (C) and without CHOL (D) in the absence of trodusquemine; $ and $$$ refer to *p* values of <0.05 and <0.001, respectively, relative to *K*_SV_ values of OAs without GM1 (C) and CHOL (D) in the presence of trodusquemine.

It is increasingly clear that membrane lipids have a crucial role in the binding of misfolded protein oligomers to the bilayer,^44−46^ particularly GM1 and CHOL.^11,39,47−49^ In order to better mimic the physiological content of GM1 and CHOL in neuronal plasma membranes, and in light of the fact that the content of these two lipids plays a crucial role in the interaction with LUVs and toxicity of misfolded protein oligomers,^11,39,44,47−50^ we decided to explore whether the variation of these two lipids in LUVs could affect the affinity of OAs for LUVs (Figure 6). To this aim, we performed the TMA-DPH quenching experiment with OAs and LUVs with 0%, 0.5%, 1%, and 5% (molar fractions) GM1 (Figure 6A). In the absence of GM1, OAs showed a significantly reduced affinity for the membrane of LUVs (*K*_SV_ value of 34.7 ± 2.0 mM^–1^), which was then found to increase with GM1 content (*K*_SV_ value up to 49.2 ± 0.4 mM^–1^ with 5% GM1), confirming the crucial role of this lipid in the membrane-oligomer interaction (Figure 6A,C). We then changed the CHOL content and performed the TMA-DPH quenching experiment with OAs and LUVs with 0%, 1%, 5%, and 10% (molar fractions) CHOL (Figure 6B). In this case, we observed a small decrease in the *K*_SV_ parameter (Figure 6B,D).

Since trodusquemine was found to preferentially bind to GM1 and CHOL in LUVs,^34^ we repeated the TMA-DPH quenching experiment using LUVs containing trodusquemine and various contents of GM1 and CHOL, in order to investigate whether the reduction of the *K*_SV_ induced by this aminosterol could be affected by the lipid composition of LUVs. Trodusquemine induced a significant reduction of the TMA-DPH fluorescence quenching at all GM1 and CHOL concentrations, with an almost complete protection from quenching at low OA concentration, and an evident quenching at higher concentration of OAs, but still lower than the corresponding values in the absence of the aminosterol (Figure 6). In the presence of trodusquemine, the *K*_SV_ value in the linear portion of the plot was found to increase with GM1 content and to slightly decrease with CHOL content, confirming the relationships observed in the absence of the small molecule (Figure 6C,D).

**Figure 7:**
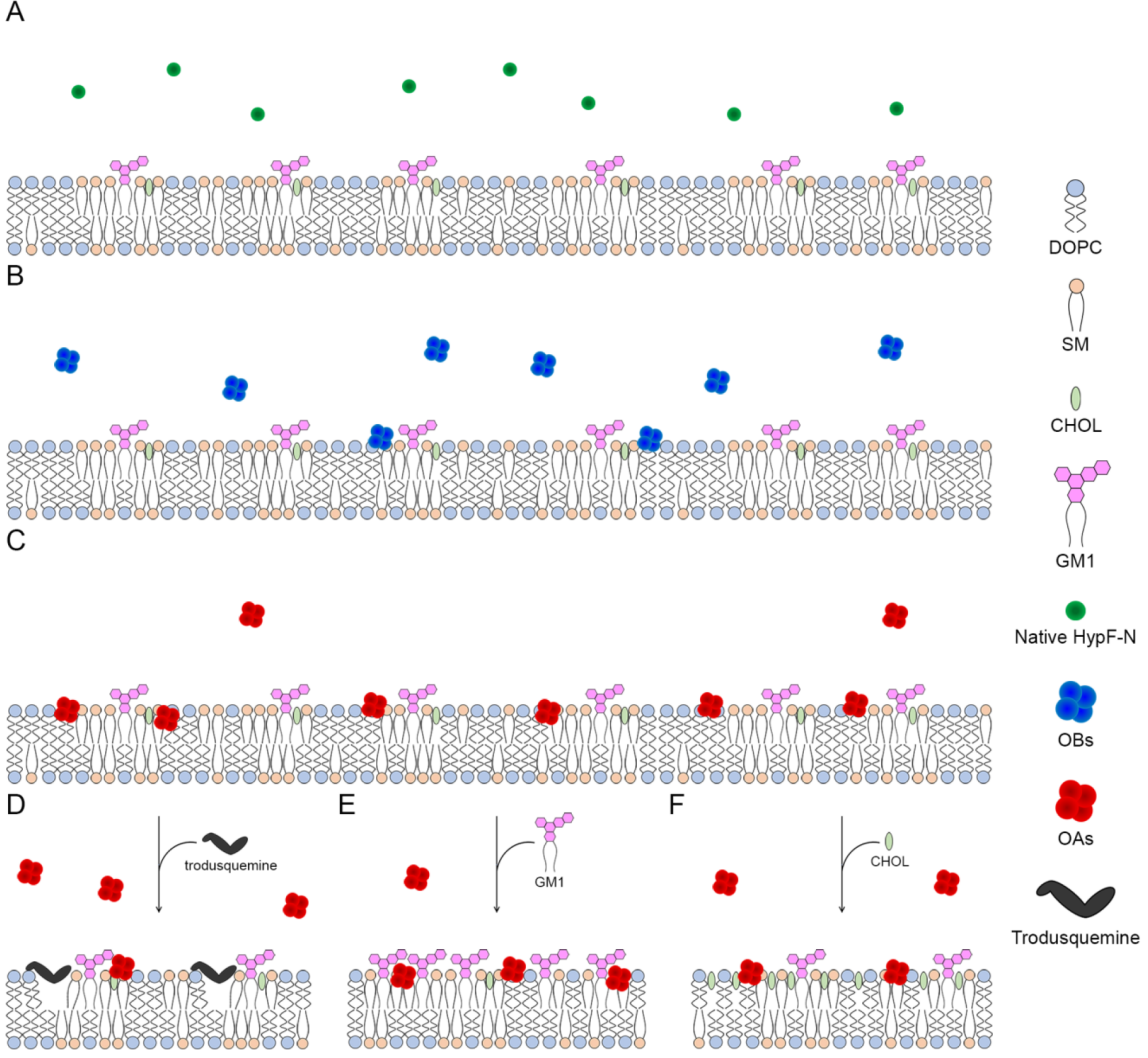
Summary of the results obtained in this work about the affinity of OAs and OBs for LUV lipid membranes. (A–C) Schematic representation of the affinity of the native protein (green, no affinity) (A), OBs (blue, low affinity) (B), and OAs (red, high affinity) (C) for the LUV lipid membrane. (D–F) Change of the OAs affinity due to the addition of trodusquemine (decreased affinity) (D), increase of GM1 concentration (increased affinity) (E), and increase of CHOL concentration (slightly decreased affinity) (F) in LUVs.

In conclusion, we have investigated quantitatively one of the main mechanisms by which toxic oligomers commonly associated with neurodegenerative diseases can exert cytotoxic effects, namely their aberrant interactions with lipid membranes. The results indicate that the toxicity of the oligomers depends on their ability to bind stably the lipid membranes, whereas nontoxic oligomers have 20–25 times reduced affinity, and native proteins do not have this action (Figure 7A–C). Correspondingly, we have also found that changes in the composition of the lipid membranes themselves, including those induced pharmacologically, can decrease the affinity of the toxic oligomers for the lipid membranes (Figure 7D–F). These results therefore offer insight on one of the fundamental molecular mechanisms of cellular degeneration caused by misfolded protein oligomers and suggest pharmacological approaches to increase the resistance of the cells to this type of insult.

## Materials and Methods

### Expression and Purification of Wild-Type and Mutant forms of HypF-N

Cultures of *E. coli* XL10 Gold harboring the pQE30-Th plasmids for the expression of wild-type HypF-N and its mutational variants^18^ were grown overnight at 37 °C under shaking in 200 mL of 20 g/L LB medium (Sigma-Aldrich, St. Louis, MO, USA) containing 100 μg/mL of ampicillin (Sigma-Aldrich). The cells were then diluted 1:20 in 4 l of fresh medium and grown at 25 °C under shaking until ∼0.6 optical density at 600 nm (OD_600_), monitored with a Jasco V-630 UV–vis spectrophotometer (Tokyo, Japan). Protein expression was induced overnight at 25 °C under shaking by the addition of 1 mM isopropyl β-d-thiogalactopyranoside (IPTG, Thermo Scientific, Waltham, MA, USA). The bacterial cells were then harvested by centrifugation for 15 min at 7000*g* at 4 °C; the pellet was resuspended in ∼30 mL of lysis buffer (50 mM phosphate buffer, 300 mM NaCl, 10 mM imidazole, pH 8.0) and stored at −20 °C overnight. The cell suspension was defrosted at 37 °C in a Thermo Haake C25P water bath (Karlsruhe, Germany) and then incubated for 1 h with 1 mg/mL lysozyme (Sigma-Aldrich) in ice under shaking followed by five cycles of sonication at 50 kHz for 30 s alternated to 30 s in ice. The cell lysate was then centrifuged for 45 min at 38 700*g* at 4 °C and the supernatant containing the protein was filtered using filters with a cutoff of 0.45 μM. The filtered supernatant was applied to an affinity chromatography column packed with HIS-Select Nickel Affinity Gel (Sigma-Aldrich), previously equilibrated with the lysis buffer at 4 °C. The column was then washed with the washing buffer (50 mM phosphate buffer, 300 mM NaCl, 20 mM imidazole, pH 8.0), equilibrated with the cutting buffer (50 mM phosphate buffer, 50 mM NaCl, pH 8.0), incubated with 50 units of human thrombin (Sigma-Aldrich) dissolved in 5 mL of cutting buffer for 1 h at 37 °C, and then incubated overnight at 4 °C under slight shaking. The pure wild-type and mutated HypF-N were then eluted using 50 mM phosphate buffer, 50 mM NaCl, 10 mM imidazole, pH 8.0. Wild-type and mutant HypF-N were then buffer-exchanged and concentrated in 5 mM acetate buffer, 2 mM dithiothreitol (DTT), pH 5.5, and in 20 mM phosphate buffer, 2 mM tris(2-carboxyethyl)phosphine hydrochloride (TCEP), pH 7.0, respectively, using an ultrafiltration cell with a 3000 Da molecular weight cutoff (MWCO) cellulose membrane (Biorad, Hercules, CA, USA) at 4 °C. The final solution containing the pure protein was centrifuged for 10 min at 16 100*g* to eliminate any aggregates and/or impurities, and its concentration was measured with a Jasco V-630 UV–vis spectrophotometer using ε_280_ = 12490 M^–1^cm^–1^. Samples were checked for their purity with SDS-PAGE and DLS, as described below, and then stored at −80 °C until use.

### SDS-PAGE Analysis of HypF-N

Samples of the various HypF-N purification steps were denatured with a 4× sample buffer (0.25 M Tris, 5.4 M glycerol, 0.3 M β-mercaptoethanol, 277 mM SDS, 6 mM bromophenol blue) and then incubated at 98 °C for 2 min. The resulting samples and a molecular weight marker (Precision Plus Protein Standard, Bio-Rad Laboratories, Hercules, CA, USA) were loaded in a precast gel with a gradient of 4–20% of acrylamide (Mini-PROTEAN TGX Precast Gels, Biorad) and then run for about 90 min at 20 mA, using the Bio-Rad Laboratories electrophoresis kit (Hercules, CA, USA). The running chambers were filled with a running buffer (25 mM Tris, 19.2 mM glycine, 0.1% SDS). The gel was then stained with Coomassie Blue dye (40% methanol, 10% acetic acid, 0.1% Coomassie Blue) for 30 min at 37 °C with slow agitation and then washed in a destaining solution (40% methanol, 10% acetic acid) for at least 1 h at room temperature to remove the excess of dye. The purified protein featured a single band at 10 kDa, in agreement with its expected molecular weight of 10 464 Da.

### DLS Analysis of HypF-N

The monomeric state of purified HypF-N was assessed acquiring its size distribution on a Zetasizer Nano S DLS device from Malvern Panalytical (Malvern, Worcestershire, UK), thermostated at 25 °C with a Peltier temperature controller and using a 10 mm reduced-volume plastic cell. The refractive index and viscosity were 1.33 and 0.89 cP, respectively. The measurement was acquired with the cell position 4.65, attenuator index 8, at 25 °C. The DLS distribution in volume mode featured a single population with a hydrodynamic diameter of 5.4 ± 1.0 nm, which is compatible with a monomeric folded HypF-N, as determined with X-ray crystallography.^51^

### Labeling of HypF-N Mutant with BODIPY FL

The C7S/C65A mutant of HypF-N (containing only Cys40) was diluted to a final concentration of 150 μM in 20 mM phosphate buffer, pH 7.0, and incubated with 2.25 mM BODIPY FL *N*-(2-aminoethyl)maleimide, previously dissolved at high concentration in dimethyl sulfoxide (DMSO) for 2 h at 25 °C in the dark on a mechanical shaker. The labeled sample was dialyzed (membrane MWCO of 3500 Da) in the dark against 1.5 l of 20 mM phosphate buffer, pH 7.0, overnight and then centrifuged to remove any precipitate. The concentration of the dye in the BODIPY FL-labeled HypF-N mutant was determined spectrophotometrically, using ε_505_ = 76 000 M^–1^ cm^–1^, whereas the concentration of the protein was determined using ε_280_ = 12490 M^–1^cm^–1^. The labeling degree of the sample was then estimated by determining the ratio between the measured dye and protein molar concentrations.

### Labeling of HypF-N Mutants with 1,5-IAEDANS and 6-IAF

The C7S/C40S/C65A/Q18C (named C18) and C7S/C40S/C65A/N34C (named C34) mutants of HypF-N (containing one cysteine residue at position 18 and 34, respectively) were labeled with 5-((((2-iodoacetyl)amino)ethyl)amino)naphthalene-1-sulfonic acid (1,5-IAEDANS) and 6-iodoacetamidofluorescein (6-IAF) dyes (Thermo Fisher Scientific, Waltham, MA, USA), respectively, as previously reported.^32^ The C18 variant was diluted to a final concentration of 180 μM in 100 mM potassium phosphate buffer, pH 7.0, with 2.7 mM 1,5-IAEDANS (15-fold molar excess of dye) and 3 M guanidine hydrochloride (GdnHCl), whereas the C34 variant was diluted to the same final concentration of 180 μM in 100 mM potassium phosphate buffer, pH 7.0, with 1.8 mM 6-IAF (10-fold molar excess of dye) and 3 M GdnHCl. Both dyes were previously dissolved in dimethylformamide (DMF) at high concentration. The two labeling mixtures were left in the dark under shaking for 2 h at 30 °C and then overnight at 4 °C. They were then dialyzed in the dark (membrane MWCO of 3000 Da) against: (i) 0.25 L of 100 mM potassium phosphate buffer, pH 7.0, with 1.5 M GdnHCl for 4 h, (ii) 0.25 L of 100 mM potassium phosphate buffer, pH 7.0 for 4 h, (iii) 0.5 L of 50 mM potassium phosphate buffer, pH 7.0, overnight, and (iv) 1.0 L of 20 mM or 5 mM potassium phosphate buffer (depending on whether the labeled mutants were used to produce type A or type B oligomers, respectively), at pH 7.0, for 6 h. The samples were then centrifuged to remove any precipitate. The concentrations of the dye in the 1,5-IAEDANS-C18 variant and in the 6-IAF-C34 variant were determined spectrophotometrically, using ε_336_ = 5700 and ε_491_ = 8200 M^–1^ cm^–1^, respectively. The protein concentration was determined spectrophotometrically using ε_280_ = 12 490 M^–1^ cm^–1^ after subtraction of the absorbance contribution of the 1,5-IAEDANS/6-IAF probe at the same wavelength of 280 nm. The labeling degree was estimated as the ratio between the two measured dye and protein molar concentrations.

### Preparation of HypF-N OAs and OBs

OAs and OBs of wild-type HypF-N were obtained at a protein concentration of 0.5 mg/mL, corresponding to 48 μM (monomer equivalents), by incubating the protein for 4 h at 25 °C in (i) 50 mM acetate buffer, 12% (v/v) trifluoroethanol (TFE), 2 mM DTT, pH 5.5 and (ii) 20 mM trifluoroacetic acid (TFA), 330 mM NaCl, pH 1.7, respectively, as previously described.^18^

BODIPY FL-labeled OAs and OBs used for the binding experiments were obtained under the same conditions, but using 4.4 μM of BODIPY FL-labeled C7S/C65A HypF-N and 43.6 μM of unlabeled C7S/C65A HypF-N, in order to obtain a 1:10 molar ratio for labeled/unlabeled protein.

OAs and OBs for intraoligomer FRET were formed under the same conditions by 24 μM C18 variant labeled with the donor dye 1,5-IAEDANS (18D) and 24 μM C34 variant labeled with the acceptor dye 6-IAF (34A), at a molar ratio 18D:34A of 1:1. These oligomers were named 18D_34A. Oligomers 18D_10 and 10_34A (with 34A and 18D replaced by the unlabeled HypF-N mutant with only one cysteine residue at position 10, respectively) were also produced in a 1:1 molar ratio between labeled and unlabeled variant.

OAs and OBs for LUV-oligomer FRET were initially formed under the same conditions using C7S/C65A HypF-N. They were then centrifuged at 16,100 g for 15 min at 20 °C. The supernatants were removed, the pellets were gently dried with nitrogen flow and then resuspended in 20 mM phosphate buffer, 2 mM TCEP, pH 7.0, to a final HypF-N concentration (monomer equivalents) of 160 μM. They were then incubated with a 12.5-fold molar excess of 1,5-IAEDANS, previously dissolved at high concentration in DMF for 2 h at 25 °C in the dark on a mechanical shaker. The labeled samples were dialyzed (membrane MWCO of 3500 Da) in the dark against 1.5 l of 20 mM phosphate buffer, pH 7.0, overnight and then centrifuged to remove any precipitate. The concentration of the dye in the samples was determined spectrophotometrically, using and ε_336_ = 5700 M^–1^ cm^–1^, whereas the concentration of the protein was determined using ε_280_ = 12 490 M^–1^ cm^–1^ after subtraction of the absorbance contribution of 1,5-IAEDANS at 280 nm. The labeling degree was estimated as the ratio between the measured dye and protein molar concentrations.

### Preparation of LUVs

LUVs were prepared using DOPC and SM in a molar ratio of 2:1 (mol/mol), 1% (mol) CHOL and 1% (mol) GM1, as previously reported.^34^ These lipid species are known to be present in the neuronal membranes.^50,52−54^ The non-natural percentages of the various lipids were chosen to favor well-separated L_β_ domains and L_α_ regions.^55^ In particular, the CHOL percentage of 1% was already adopted in other works^30,34,55−58^ and it is necessary to obtain model membranes with well distinct L_α_ and L_β_ phases and relatively extended L_β_ domains. It was demonstrated that the L_β_ domains significantly decreased their size by increasing the CHOL concentration, becoming indistinguishable for microscopic analyses.^55^ The ordered domains of LUVs, enriched in GM1 and CHOL, partially mimic the complex features of lipid rafts in neurons.^50,59^ Moreover, the progressive increase of CHOL and GM1 content in our experiments, aim at better mimicking the real composition of neuronal membranes.

LUVs were obtained by dissolving the desired lipid mixture in chloroform/methanol (2:1) and by removing the organic solvent by evaporation in vacuo (Univapo 150H, UniEquip, Munich, Germany) for 3 h. The mixtures were hydrated with distilled water to form multilamellar vesicles (MLVs) to a total lipid concentration of 2 mg/mL for quenching experiments, 3.5 mg/mL for binding experiments, 1 mg/mL for LUV-oligomer FRET experiments, and 3 mg/mL for circular dichroism (CD), tryptophan fluorescence and intraoligomer FRET experiments (mother solutions). MLVs were left to swell for 1 h at 60 °C and then extruded 17 times through a polycarbonate membrane with 100 nm pores using a mini-extruder (Avanti Polar Lipids) at the same temperature, to form LUVs. After cooling to room temperature, LUVs were stored at 4 °C for a maximum of 1 week. The lipid mixtures were: (i) 1,2-dioleoyl-*sn*-glycero-3-phosphocoline (DOPC, Avanti Polar Lipids, Alabaster, AL, USA) and sphingomyelin (SM, Sigma-Aldrich, Darmstadt, Germany) in a molar ratio of 2:1 (mol/mol), 1% (mol) cholesterol (CHOL, Sigma-Aldrich) and 1% (mol) monosialotetrahexosylganglioside 1 (GM1, Avanti Polar Lipids); (ii) DOPC and SM in a molar ratio of 2:1 (mol/mol), 1% (mol) CHOL; (iii) DOPC and SM in a molar ratio of 2:1 (mol/mol), 1% (mol) CHOL and 5% (mol) GM1; (iv) DOPC and SM in a molar ratio of 2:1 (mol/mol) and 1% (mol) GM1; (v) DOPC and SM in a molar ratio of 2:1 (mol/mol), 5% (mol) CHOL and 1% (mol) GM1; (vi) DOPC and SM in a molar ratio of 2:1 (mol/mol), 10% (mol) CHOL and 1% (mol) GM1. LUVs containing trodusquemine were obtained by adding the aminosterol during the hydration phase to obtain final trodusquemine and total lipid concentrations of 5 μM and 0.3 mg/mL, respectively.

### Interaction between OAs/OBs and SLBs Using AFM

The interaction was tested on SLBs with three different lipid mixtures: (i) DOPC/SM 2:1 (mol/mol), 1% (mol) CHOL; (ii) DOPC/SM 2:1 (mol/mol), 1% (mol) CHOL, 1% (mol) GM1; (iii) DOPC/SM 2:1 (mol/mol), 1% (mol) CHOL, 5% (mol) GM1. Aliquots (40 μL) of LUV suspensions (0.1 mg/mL) were deposited onto a 1.0 × 1.0 cm^2^ freshly cleaved mica substrate with 10 μL of a 10 mM CaCl_2_ solution. The samples were kept for 15 min at room temperature and then incubated for 15 min at 60 °C in a close chamber with 100% relative humidity to form a uniform SLB. Subsequently, the samples were cooled at room temperature and gently rinsed three times with Milli-Q water. A Nanowizard III (JPK Instruments, Germany) mounted on an Axio Observer D1 (Carl Zeiss, Germany) inverted optical microscope was used to acquire the AFM images. V-shaped DNP silicon nitride cantilevers (Bruker, MA, USA), with a typical tip curvature radius of 20–60 nm, nominal spring constant 0.24 N/m, and a resonance frequency in air ranging from 40 kHz to 75 kHz were used. The measurements were carried out in water using the intermittent contact mode in the constant-amplitude mode, working with an oscillating frequency of 10–20 kHz. The amplitude set point was kept above 70% of free oscillation amplitude in all cases. OAs and OBs were administered under the AFM head at a final concentration of 12 μM and left standing to interact with the SLBs for 30 min. AFM images (512 × 512 image data points) were processed using the JPK Data Processing software (JPK Instruments, Germany). The difference in thickness (Δ*Z*) between gel (L_β_) and fluid (L_α_) lipid domains was determined by considering image height distributions. The distributions were fitted to the sum of two Gaussian functions, and the Δ*Z* value was determined as the difference between the peaks of the two Gaussian functions. This procedure was repeated for at least 10 different images for each experiment.

### Intrinsic Tryptophan Fluorescence Assay

OAs, OBs, and native HypF-N were diluted in 20 mM phosphate buffer, pH 7.0, in the presence of different concentrations of LUVs (0, 0.04, 0.08, 0.12, 0.24, 0.30, 0.50, 1.00, 1.50 mg/mL) to a final HypF-N concentration (monomer equivalents) of 1.9 μM (OAs and OBs) and 20 μM (native HypF-N) for 15 min at 25 °C. DLS was used to assess the integrity of LUVs upon change of solution conditions from distilled water (in which they were prepared) to 20 mM phosphate buffer, pH 7.0. The structural integrity of OAs and OBs upon change of solution conditions was assessed previously.^18^ Intrinsic tryptophan fluorescence spectra were then acquired at 25 °C from 300 to 450 nm (excitation at 280 nm) using a 3 mm × 3 mm black wall quartz cell on a PerkinElmer LS 55 spectrofluorimeter (Wellesley, MA, USA) equipped with a thermostated cell-holder attached to a Haake F8 water-bath (Karlsruhe, Germany), or on an Agilent Cary Eclipse spectrofluorimeter (Agilent Technologies, Santa Clara, CA, USA) equipped with a thermostated cell holder attached to a Agilent PCB 1500 water Peltier system.

### Far Ultraviolet (Far-UV) CD

OAs, OBs and native HypF-N were diluted in 20 mM phosphate buffer, pH 7.0, in the presence of different concentrations of LUVs (0, 0.04, 0.08, 0.12, 0.24, 0.30, 0.50, 1.00, 1.50 mg/mL), to a final HypF-N concentration (monomer equivalents) of 20 μM for 10 min at 25 °C. The far-UV CD spectra were collected over the 190–260 nm wavelength range at 25 °C using a using a 1 mm path length cell on a Jasco J-810 spectropolarimeter (Tokyo, Japan) equipped with a thermostated cell holder attached to a Thermo Haake C25P water bath (Karlsruhe, Germany). All spectra were truncated at HT > 700 V, blank-subtracted and normalized to mean residue ellipticity using
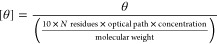
1where [θ] is the mean residue ellipticity in deg cm^2^ dmol^–1^, θ is the ellipticity in mdeg, optical path is in cm, concentration is in g/l, and molecular weight is in g/mol.

### Intraoligomer FRET

OAs and OBs (18D_34A, 18D_10 and 10_34A) formed at a total monomer concentration of 48 μM were diluted in 20 mM and 5 mM potassium phosphate buffer (for OAs and OBs, respectively), pH 7.0, to obtain a final HypF-N concentration (monomer equivalents) of 20 μM, and in the presence of different concentrations of LUVs (0.12, 0.3, 0.5, and 0.7 mg/mL) prepared as described above. Oligomers and LUVs were incubated for 10 min at 25 °C. The samples were diluted to a final HypF-N concentration of 2 μM immediately before fluorescence acquisition. Fluorescence emission spectra were recorded on a PerkinElmer LS55 spectrofluorimeter (Wellesley, MA, USA) equipped with a thermostated cell-holder attached to a Haake F8 water-bath (Karlsruhe, Germany). The measurements were performed using a 2 mm × 10 mm quartz cell at 25 °C with excitation at 336 nm. The FRET efficiency (*E*) values between 18D and 34A in OAs and OBs were calculated as
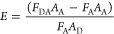
2where *A*_A_ and *A*_D_ represent the absorbance values at 336 nm of acceptor (*A*_*A*_ = 0.05) and donor (*A*_D_ = 0.07), respectively, obtained in the presence of a concentration of dye of 120 μM; *F*_*DA*_ and *F*_*A*_ represent the acceptor fluorescence emission at 1 mM (excitation 336 nm) obtained in the presence and in the absence of donor, respectively, determined from the area between 490 and 600 nm below the corresponding curves.^32^

### LUVs-Oligomers FRET

LUVs were prepared at a total lipid concentration of 1 mg/mL, as described above, in the presence of either BODIPY-FL C5-ganglioside GM1 (GM1-A, commercial name BODIPY-FL C5-Ganglioside GM1, ThermoFisher Scientific), BODIPY-FL cholesterol (CHOL-A, commercial name TopFluor cholesterol, Avanti Polar Lipids), BODIPY-FL-sphingomyelin (SM-A, commercial name TopFluor Sphingomyelin, Avanti Polar Lipids) or BODIPY-FL-DOPC (DOPC-A, commercial name TopFluor PC, Avanti Polar Lipids) used as acceptors, with a molar fraction of each labeled lipid of 1% relative to total lipids in all cases. OAs from the C7S/C65A mutant labeled on their surface with 1,5-IAEDANS were prepared at a total protein concentration of 160 μM, as described above, and used as donor (OA-D). Fluorescence spectra of 0.3 mg/mL nonlabeled LUVs incubated with 20 μM OA-D (OA-D spectra), 0.3 mg/mL LUVs containing lipid-A incubated with 20 μM nonlabeled OAs (Lipid-A spectra), and 0.3 mg/mL LUVs containing lipid-A incubated with 20 μM OA-D (OA-D+Lipid-A spectra) were acquired after 15 min of incubation on a PerkinElmer LS 55 spectrofluorimeter equipped with a thermostated cell-holder attached to a Haake F8 water bath. The spectra were acquired from 350 to 600 nm using a 3 × 3 mm black wall quartz cell at 25 °C, with excitation at 336 nm. The FRET *E* was calculated as
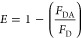
3where *F*_DA_ is the fluorescence intensity of D in the presence of A and *F*_D_ is the fluorescence intensity of D in the absence of A.

### Fluorescence Quenching of DPH and TMA-DPH in LUVs

1,6-Diphenyl-1,3,5-hexatriene (DPH, Sigma-Aldrich) and 1-(4-trimethylammoniumphenyl)-6-phenyl-1,3,5-hexatriene p-toluenesulfonate (TMA-DPH, ThermoFisher scientific) were dissolved in chloroform/methanol (2:1) and added to the lipid mixture to obtain a probe:lipid molar ratio of 1:300. LUVs were then prepared at 2 mg/mL as described above, diluted with distilled water to 0.3 mg/mL and incubated with increasing concentrations of OAs, OBs, and native protein (0 to 32.5 μM) at 25 °C for 15 min in the dark. The fluorescence spectra of the resulting samples were acquired at 25 °C from 380 to 550 nm (excitation 355 nm) using a 3 mm × 3 mm black walls quartz cell on an Agilent Cary Eclipse spectrofluorimeter (Agilent Technologies, Santa Clara, CA, USA) equipped with a thermostated cell holder attached to an Agilent PCB 1500 water Peltier system. The quenching of TMA-DPH and DPH was then analyzed with the Stern–Volmer equation
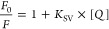
4where *F*_0_ and *F* are the integrated fluorescence intensity areas at 400–500 nm in the absence and presence of the quencher (OAs, OBs, or native proteins), respectively; [Q] is the concentration of the quencher and *K*_SV_ is the Stern–Volmer constant. The plot of quenching of TMA-DPH and DPH in 0.3 mg/mL LUVs containing 5 μM trodusquemine was analyzed from 12.5 μM OAs with an equation derived from the Stern–Volmer equation
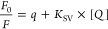
5where *q* is the intercept and all the other parameters have the same meaning as in eq 4.

### Binding Assay of OAs, OBs and Native Proteins to LUVs

OAs, OBs and native HypF-N formed by BODIPY FL-labeled and unlabeled HypF-N C7S/C65A mutant (molar ratio 1:10) were diluted in 20 mM phosphate buffer, pH 7.0, to obtain a final HypF-N mutant concentration of 20 μM (monomer equivalents), and were incubated for 15 min at 25 °C, with increasing concentrations (from 0 to 2.0 mg/mL) of LUVs prepared as described above. In experiments involving trodusquemine-containing LUVs, the concentration of the small molecule was variable but the trodusquemine/lipid molar ratio was maintained and corresponded to 5 μM in 0.3 mg/mL LUVs. The fluorescence spectra were acquired at 25 °C from 490 to 560 nm (excitation 480 nm) using a 3 mm × 3 mm black walls quartz cell on the Agilent Cary Eclipse spectrofluorimeter described above. The fluorescence emission at 512 nm was then plotted versus LUV concentration and analyzed with
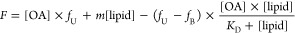
6where *F* is the observed fluorescence at 512 nm, [OA] is the molar concentration of OAs (monomer equivalents), *f*_U_ and *f*_B_ are the fluorescence emission of the unbound and bound OAs at unitary concentration of OAs, respectively, *m* is the dependence of *F* on [lipid] after binding (drift), [lipid] is the molar concentration of total lipids in LUVs, and *K*_D_ is the dissociation constant.

## Supporting Information Available

AFM images of SBLs containing 0, 1, 5% GM1 treated with OAs and OBs; Δ*Z* measured on SLBs with different GM1 contents; magnification of TMA-DPH and DPH Stern–Volmer plots; binding plot of GSH labeled with BODIPY-FL; chemical structure of trodusquemine (PDF)
