# Supplementary material for: Quantitative Measurement of the Affinity of Toxic and Nontoxic Misfolded Protein Oligomers for Lipid Bilayers and of its Modulation by Lipid Composition and Trodusquemine
Source: ACS Chem Neurosci. 2021 Aug 12;12(17):3189–202. doi: 10.1021/acschemneuro.1c00327 (PMC8414483; doi:10.1021/acschemneuro.1c00327)
Supplement: Supplementary file 1 — cn1c00327_si_002.pdf [file cn1c00327_si_002.pdf]

Supporting Information

Quantitative Measurement of the Affinity of Toxic and Nontoxic Misfolded Protein Oligomers for Lipid Bilayers, and of its Modulation by Lipid Composition and Trodusquemine

Silvia Errico, [a,b] Hassan Ramshini,[a,c] Claudia Capitini, [d,e] Claudio Canale,[f] Martina Spaziano,[a] Denise Barbut,[g] Martino Calamai,[d,h] Michael Zasloff,[g,i] Reinier Oropesa-Nuñez,[l] Michele Vendruscolo,[b] Fabrizio Chiti[a*]

[a] *Department of Experimental and Clinical Biomedical Sciences, Section of Biochemistry, University of Florence, Florence 50134, Italy*

[b] *Centre for Misfolding Diseases, Yusuf Hamied Department of Chemistry, University of Cambridge, Cambridge CB2 1EW, UK*

[c] *Department of Biology, Payame Noor University, Tehran 19395-4697, Islamic Republic of Iran*

[d] *European Laboratory for Non-linear Spectroscopy (LENS), Sesto Fiorentino 50019, Italy*

[e] *Department of Physics and Astronomy, University of Florence, Sesto Fiorentino 50019, Italy*

[f] *Department of Physics, University of Genoa, Genoa 16146, Italy*

[g] *Enterin Inc., 2005 Market Street, Philadelphia, Pennsylvania 19103, United States*

[h] *National Institute of Optics, National Research Council of Italy (CNR), Florence 50125, Italy*

[i] *MedStar-Georgetown Transplant Institute, Georgetown University School of Medicine, Washington DC 20007, USA*

[l] *Department of Materials Science and Engineering, Uppsala University, Uppsala SE-751 03, Sweden*

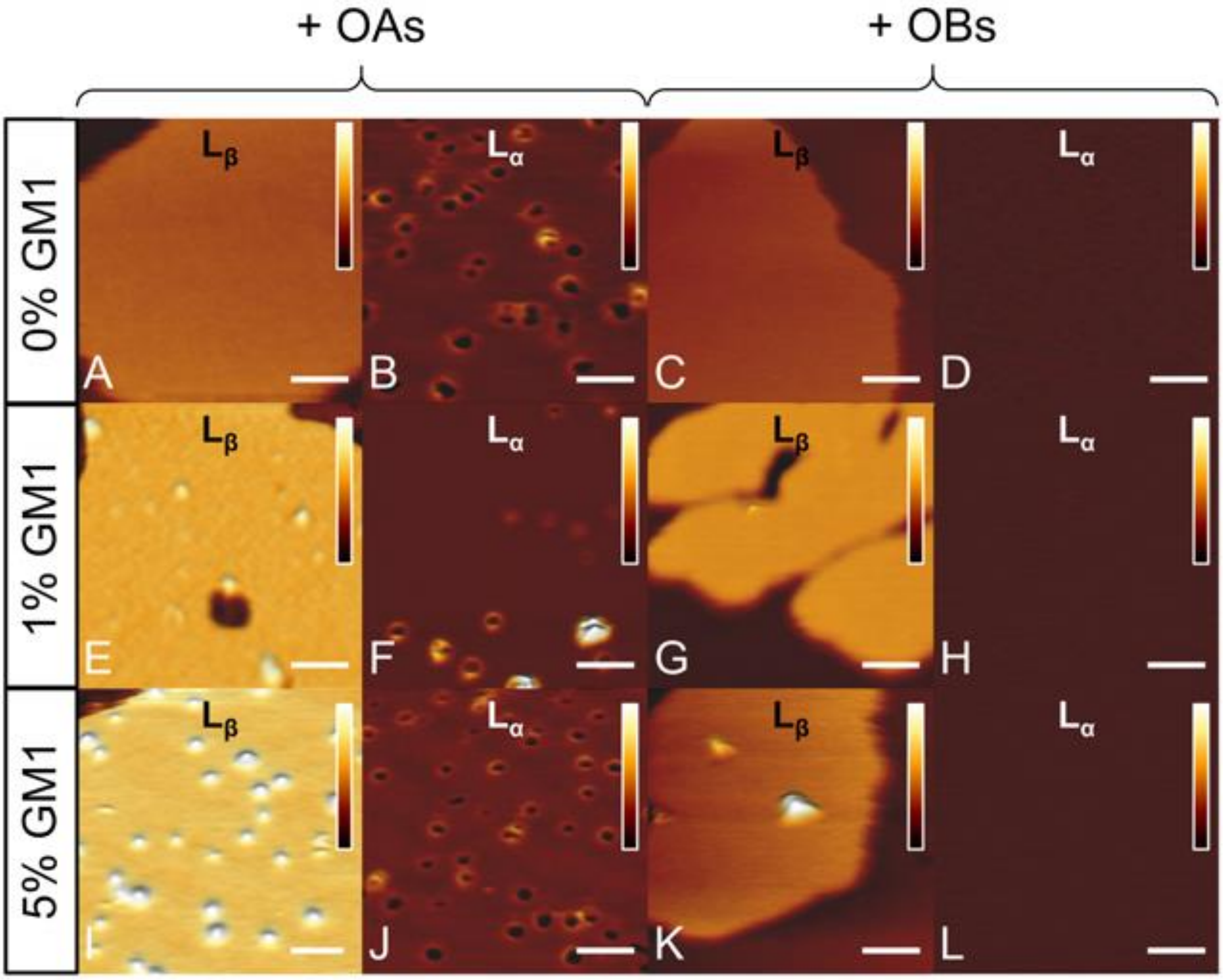

Figure S1. Tapping mode AFM images of SLBs treated with OAs **(A,B,E,F,I,J)** and with OBs **(C,D,G,H,K,L)**. The concentration of GM1 varied from 0% **(A-D)**, to 1% **(E-H)** and 5% **(I-L)** molar fraction. OAs bind the L_β ordered phase domains in the presence of GM1 (**E,I**), whereas the binding for OBs involves a lower number of oligomers (**G,K**). Neither OAs nor OBs are binding the L_β ordered phase domains in the absence of GM1 (**A,C**) and the number of oligomers on SLBs depends on the GM1 concentration. Toxic OAs also form annular structures on the disorder L_α phase domains **(B,F,J),** but these were not found to correlate with oligomer toxicity (Oropesa-Nuñez et al. 2016). The interaction between OBs and SLBs is weak in all cases **(C,D,G,H,K,L)**. Scale bars: 100 nm. Vertical color scale: 5 nm.

Table S1. ΔZ measured on SLBs with different GM1 contents. The values obtained for untreated samples are compared with those after treatment with OAs or OBs. The modification of the bilayer structure induced by OAs is reflected in the variation of ΔZ.

Lipid composition (molar fraction)	ΔZ (nm)		
	Untreated	Treated with OAs	Treated with OBs
0% GM1	1.1±0.3	2.2±0.1	1.0±0.1
1% GM1	2.0±0.3	2.8±0.1	2.1±0.1
5% GM1	2.1±0.1	3.7±0.1	2.2±0.1

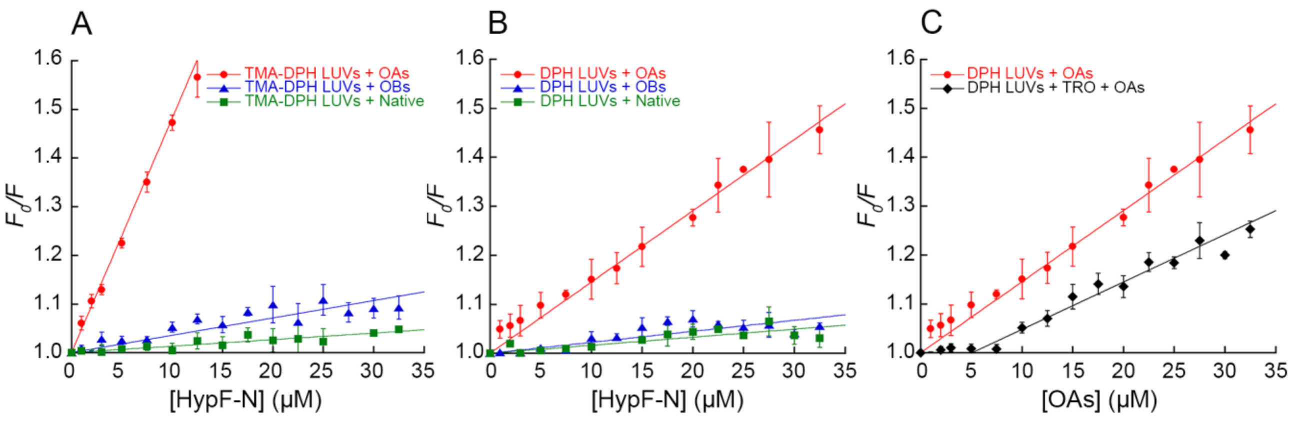

Figure S2. (A,B) Magnification of the Stern-Volmer plots reported in Fig.1A,B, showing the ratio of fluorescence of TMA-DPH (A) and DPH (B) in 0.3 mg/ml LUVs in the absence (F_0) or presence (F) of various concentrations (monomer equivalents) of OAs (red circles), OBs (blue triangles) and native HypF-N (green squares). The straight lines through the data points represent the best fits to Eq. 4. Experimental errors represent s.e.m. of 2-5 experiments. **(C)** Magnification of the Stern-Volmer plot reported in Fig. 5B, showing the ratio of fluorescence of DPH in the absence (F_0) or presence (F) of various concentrations (monomer equivalents) of OAs, in the absence (red circles) and presence (black diamonds) of 5 µM trodusquemine (TRO) in 0.3 mg/ml LUVs. The straight lines through the data points represent the best fits to Eq. 6 (red line) and Eq. 7 (black line). Experimental errors represent s.e.m. of 2-5 experiments.

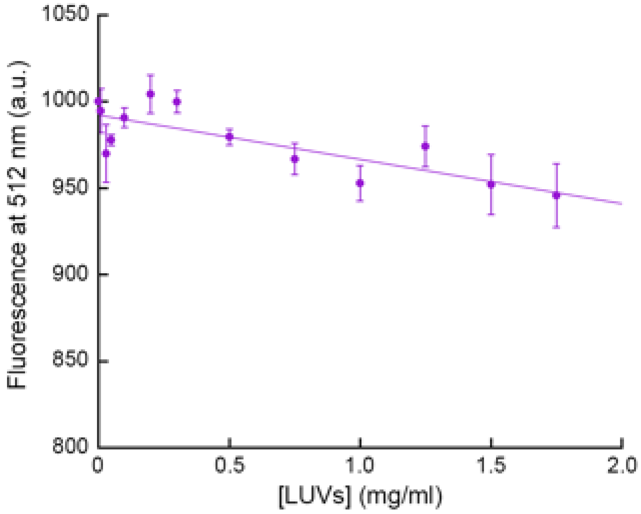

Figure S3. Binding plot reporting the fluorescence at 512 nm of 20 µM GSH labelled with BODIPY FL *versus* LUV concentration. The line through the data points represents the best fits to a linear function. Experimental errors represent s.e.m. of 3 experiments.

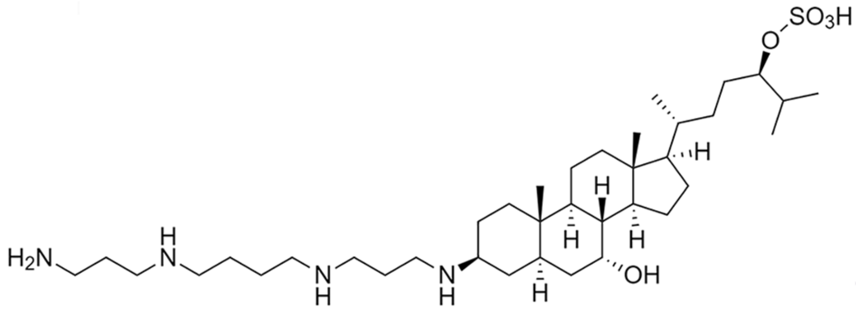

Figure S4. Chemical structure of trodusquemine. The molecule consists of a steroid moiety of the type of cholestane, with hydroxyl and sulfate groups at C-7 and C-24, respectively, coupled to a spermine moiety at C-3.
